# ‘Remoteness was a blessing, but also a potential downfall’: traditional/subsistence and store-bought food access in remote Alaska during the COVID-19 pandemic

**DOI:** 10.1017/S1368980023000745

**Published:** 2023-04-18

**Authors:** Ruby L Fried, Micah B Hahn, Patricia Cochran, Laura P Eichelberger

**Affiliations:** 1 Institute for Circumpolar Health Studies, University of Alaska Anchorage, 1901 Bragaw, Suite 220, Anchorage, AK 99508, USA; 2 Alaska Native Science Commission, Anchorage, AK, USA; 3 Alaska Native Tribal Health Consortium, Research Services, Tribal Water Center, Anchorage, AK, USA

**Keywords:** Food security, Rural, COVID-19, Arctic

## Abstract

**Objective:**

This study employs a strengths-based approach to assess food access in remote Alaska during the COVID-19 pandemic, identifying both the negative consequences of the pandemic on store-bought and subsistence/traditional food access and compensatory strategies used.

**Design:**

As a part of a larger study on the impacts of COVID-19 on daily life remote Alaskan communities, study data presented here were collected through key informant interviews (KII) and state-wide online surveys from 21 September 2020 to 31 March 2021 among remote Alaska community members.

**Setting:**

This study was conducted with residents of remote communities in Alaska, defined as those off the road system. Remote communities often have small or no grocery stores and rely on subsistence or traditional sources of food.

**Participants:**

KII participants (*n* 36) were majority female (78 %) and Alaska Native (57 %). Survey participants (*n* 615) were also majority female, 25–54 years old and most had had some post-secondary education or training.

**Results:**

Survey and interview data revealed that the pandemic had significant negative impacts on store-bought food access in remote Alaskan communities. Individuals also shared that locally available and wild harvested foods acted as a buffer to some of the loss of access to these store-bought foods, with some people sharing that the harvesting of wild and traditional foods served as a coping strategy during times of pandemic-related stress.

**Conclusions:**

The results from this study demonstrate that the remoteness of some Alaskan communities has been both a source of vulnerability and protection in terms of food access.

The COVID-19 pandemic has disrupted political, economic and social patterns, in many cases revealing and exacerbating existing racially and geographically based inequities and vulnerabilities in supply chains^(1,2)^. Food access and security are no exception, with panic shopping, increased food prices, effects on infrastructure and transportation, more limited access to food assistance programmes and decreases in employment negatively impacting food access for many individuals and families in the USA and beyond^(3,4)^. Early data from the pandemic demonstrated increase in household food insecurity of up to 30–38 %, depending on population^(5–7)^, and a steep increase among rural Indigenous communities in the USA because of limited food supplies at local grocery stores and food banks^(8)^.

High levels of food insecurity have been found among remote many Arctic populations, with reported incidences as high as 56–83 %, significantly exceeding that of urban and peri-urban areas^(9,10)^. Existing food access inequalities can be attributed to more limited access to market-based employment, forced acculturation, geographic isolation and climate change in rural and remote Arctic communities^(11–13)^. Food access is of paramount concern in Alaska as well, with over 13 % of households being food insecure across the state and an even higher proportion in rural areas^(14,15)^. There are 60 000 people living in remote Alaskan communities, many of which are accessible only by plane year-round. Remote Alaska is characterised by ‘hub’ and ‘spoke’ or village communities, the former having larger populations and greater access to transportation, food, health care and other services. Smaller villages often have small stores or no grocery store, and many community members rely on the postal service, trips to hub or urban communities for large-scale food purchases and subsistence or traditional sources of food through hunting, fishing and harvesting greens and berries. Residents of rural (both hub and village) communities also engage in subsistence/traditional harvesting practices, with harvests averaging about 295 pounds per person in remote Alaska, in comparison to just 22 pounds per person in urban areas^(16)^. Due to the importance to wild foods, scholars and leaders have called for greater inclusion of measures of traditional/subsistence food access in order to characterise food security more accurately, particularly in areas with high Indigenous representation^(17,18)^.

During the COVID-19 pandemic, the remoteness of these communities has been both a source of vulnerability and protection. Many communities were able to limit entry to their communities early in the pandemic and were initially able to remain infection free. From the beginning of the pandemic, many remote communities across Alaska restricted travel and implemented ‘hunker down’ orders, requiring residents to shelter in place and only allowing for essential or very limited travel with required quarantine upon arrival to the community^(19)^. Restricted travel, including waiting periods and proof of negative tests, was in place during the study period in many remote communities, regardless of vaccination status of travellers. Due to their geographic isolation and community leadership, most of remote Alaska did not experience community spread of SARS-Cov2 infection until June 2020 compared with urban areas that had cases as early as March 2020^(20)^.

However, despite a relatively small number of COVID-19 cases in remote communities, by March 2020 the pandemic was contributing to limited access of goods and services because of reduced travel, postal services and the reduced frequency and volume of cargo and transportation services. Particularly relevant to store-bought food access, reduced and cancelled airline service contributed to severe disruptions in food supply chains of both fresh and shelf-stable goods^(21)^. This was further exacerbated by the ceasing of operations and declaration of bankruptcy of one Alaska-based air carrier and lifeline for goods and services, in early April 2020^(22)^. The State of Alaska Marine Highway System, a ferry system that is vital for access to food and medical care, also dramatically cut back its already-reduced service to isolated communities in the Southeast and Gulf Coast regions^(23)^. While store-bought food became increasingly more difficult to access in many remote communities, local subsistence/traditional food harvesting persisted in many areas, but not without limitations, discussed below.

In this study, we use a strengths-based approach^(24)^ to assess food access in remote Alaska during the pandemic, identifying both the negative consequences of the COVID-19 pandemic on store-bought and subsistence/traditional food access and coping strategies that people have used to deal with inconsistent food access. The data presented here were collected as a part of a larger study on the impacts of COVID-19 on remote Alaskan communities.

## Methods

### Study population and participants

The study population were adults 18 years and older who were residents of remote Alaskan communities at the time of data collection. We defined remote Alaskan communities as those off the road system, excluding Juneau, Alaska due to its large population (> 30 000 people) and relatively greater ease of access to services and amenities. Survey participants (*n* 615) were residents of remote Alaskan communities, and key informant interview (KII) participants (*n* 36) were survey respondents who had self-selected for a follow-up interview.

### Study design

Data collection included two rounds of surveys as well as KII following the surveys. We conducted two waves of surveys in order to capture the impacts of COVID-19 on various aspects of daily life before (survey 1: 9 November–15 December 2020) and after (survey 2: 9–25 March 2021) the availability and distribution of COVID-19 vaccines to all individuals aged 16 years or older.

Survey and KII questions were developed with the study’s Elder advisor (Cochran) and five other advisors who were leaders and healthcare providers who lived and/or worked in remote Alaskan communities. Each advisor reviewed the interview and survey questions and provided feedback through meetings with the study team on wording, flow and overall approach based on their long history and pandemic-related interactions with community members. The wording and overall design of the instruments were revised according to this feedback prior to data collection.

Survey participants were recruited through Facebook and community contacts. Survey data were collected using REDCap. Remote Alaska residency was confirmed by participants providing their zip code. Records indicating zip codes in non-remote communities removed from the study sample prior to analysis. KII were conducted in order to provide additional, in-depth information about how COVID-19 had impacted food security beyond what captured in the online surveys.

This study was conducted according to the guidelines laid down in the Declaration of Helsinki, and all procedures involving research study participants were approved by the Alaska Area Institutional Review Board on 26 June 2020 (Protocol: 1590924-8; IRB Reference #: 2020-05-021-8) and the Yukon-Kuskokwim Health Corporation’s Full Board of Directors on 18 August 2020. Written informed consent was obtained from all survey participants, and verbal informed consent was obtained from all KII participants.

### Data collection

Study data were collected through KII (21 September 2020–31 March 2021) and two rounds of state-wide online surveys of remote Alaska community members (survey 1: 9 November–15 December 2020; survey 2: 9–25 March 2021). With a population in remote Alaskan communities of ∼166 000 (population estimate, 2019), we expected a margin of error of 9·5 % and with a sample size of 108 (survey 1) and of 4·3 % with a sample size of 508 (survey 2).

The online study survey took participants 15–20 min to complete and had multiple choice and short-answer questions that were about the impacts COVID-19 had on daily life in remote communities, including those regarding food access. Participants were provided the following prompt: ‘Please tell us about your family’s experiences during the COVID-19 pandemic. When answering these questions, please think about what has happened from late winter (March 2020) to the present, due to COVID-19’. Survey data regarding food access were subsequently collected using YES/NO responses to the statements, ‘We had difficulty getting food from the store’, ‘We had difficulty getting subsistence/traditional foods’ and ‘We had difficulty paying for groceries or utilities’.

In addition, survey participants were asked whether they had spent time engaging in traditional/subsistence activities, eaten high fat or sugary foods, eaten more food and/or eaten less food in order to cope with the COVID-19 pandemic. Survey data also included demographic characteristics such as sex/gender, age, race/ethnicity, educational attainment and annual household income.

Due to COVID-19 travel and gathering restrictions, individuals participated in the study through a semi-structured telephonic KII and/or an online survey. KII data regarding food access were obtained from open-ended interview questions including: ‘What are the problems or issues that your community is facing due to COVID?’, as well as more food-specific questions such as ‘Has the pandemic affected your ability to get foods from the store? If yes, how did you get food?’ and ‘How has the pandemic affected your ability to get and share traditional/subsistence foods?’.

### Data analysis

We tested whether there were significant differences in the demographic characteristics of participants of survey rounds 1 and 2 using *t* tests, chi-square and fisher exact tests using SAS, Version 9.4 (SAS Institute, INC.). Demographics tested included age, sex, annual household income, educational attainment and race/ethnicity. Survey data were corrected for response bias in this non-probability-based sample using post-stratification weighting. To construct post-stratification weights, we utilised population data from the Alaska Department of Labor and Workforce development that included population estimates based on census data, including age, sex and race. These data were used to adjust survey responses to match the demographic distribution of remote Alaska residents more closely. Summary statistics from survey data were produced using SAS, Version 9.4 (SAS Institute, INC.).

KII responses were double coded by two individuals using inductive coding of themes. The two coders then met to reconcile any differences between themes, and interviews were re-coded according to the finalised codebook. Codes are not mutually exclusive.

## Results

### Key informant interviews community members

We have listed each response or theme in addition to enumerating each of the thirty-six KII responses, to mitigate strong implications of hierarchy in importance and/or frequency of response. The majority of respondents were female, between the ages of 30–59 years old, with a slight majority identifying as Alaska Native (Table 1).

**Table 1:** Key informant demographic characteristics

Demographic characteristic	Number of respondents (*n* 36)	Percentage total
Sex		
Male	8	22
Female	28	78
Age (years)		
18–29	5	14
30–59	24	66
60+	7	20
Race/ethnicity		
Alaska Native	21	57
Non-native	15	43

#### COVID-19-related impacts on store-bought food access

Impacts on store-bought food access were cited in twenty-five of thirty-six total interviews (70 %). A total of eleven interviews (31 %) (not included in Table 2) also cited impacts on food access, which did not specify store-bought or traditional/subsistence foods. Specific themes that arose during KII are detailed below (Table 2), which outlines both positive and negative COVID-19-related impacts on store-bought food access.

**Table 2:** Positive and negative COVID-19-related impacts on store-bought food access identified by key informants

Positive impact on store-bought food access	Code definition	Discussed in # of interviews (*n* 36)	%
Food assistance	Food access through food assistance, including churches, schools and local food banks	8	22
Delivery/phone orders	Food access is made easier by stores offering delivery and/or phone-order services	8	22
Going to the store for people in quarantine	Other community members helping with food access by shopping for individuals in quarantine	6	17
Negative impact on store-bought food access	Code definition	# (%) of interviews	
Travel/transportation restrictions	Restrictions on travel/transportation to the remote community from urban and other remote communities reduced capacity to obtain foods in general (*n* 14), through air travel (*n* 5), through ferry service (*n* 2) and to obtain less expensive store-bought foods (*n* 3)	24	66
Limited food available at store	Reduced access due to less food being available at the local store, both variety and amount	6	17
Disruption in postal service/shipping	Reduced access due to disruption in postal service/shipping services, including postal office closure and reduced shipping/freight services	6	17
Reduced access to store	Reduced access to store due to hunker down orders, store closure, limited hours, limited hours for certain groups (Elders, certain houses, etc.)	5	14
Increased food prices	Increased food prices during the pandemic	5	14
No local grocery store	Absence of local grocery store	3	8
Panic shopping	Reduced access to food due to other community members ‘panic shopping’ and lack of supplies to replace food items	2	6

Store-bought food access was impacted positively by communities and businesses responding with food assistance, changes in grocery store operations and community members shopping for others when they were required to be quarantined. For example, one interviewee summarised these findings in stating that, ‘Grocery stores are delivering, food bank is delivering, people [are] helping out Elders, making sure people have someone to help with groceries’. Another respondent who works in health care and is an Elder specifically connected public health responses to store-bought food access:

I’m really proud of [my community] - in how they implemented all their public health. They had these sections of houses that will go to the store at these hours, or in the mornings they reserved one hour for their Elders to go to the store in each group of houses…And if an Elder couldn’t go to the store, [or] if a house had COVID, then someone went to the store for them.

However, travel restrictions, increased food prices and disruptions in postal services/shipping negatively impacted store-bought food access in remote communities. Referencing restricted travel to hub and large urban communities, one respondent expressed frustration: ‘[We] haven’t been able to “grub up” - have to quarantine for 2 weeks. If someone wants to get a box of macaroni that doesn’t cost $7, that’s going to cost you two weeks. We’ve been shopping online’. Another interviewee, a woman living in remote community in southeast Alaska, cited the vulnerability of supply chains, panic shopping and delayed shipping as additional difficulties to obtaining store-bought foods in their community:

The volatile food-based market…can be scary in a rural community…The people who could stock up and panic shop did, and that left shelves bare…A lot of people here utilized Amazon prime because of their free shipping - but the service delivery was really varied - could get it right away or wait up to 6 weeks.

#### COVID-19-related impacts on traditional/subsistence food access

Impacts on traditional/subsistence food access were cited in twenty-three of thirty-six total interviews (64 %). While most respondents discussed specific positive or negative impacts of the COVID-19 pandemic on traditional/subsistence food access (Table 3), seven interviews (19 %) reported that the pandemic had no impact on access to these foods.

**Table 3:** Positive and negative COVID-19-related impacts on traditional/subsistence food access (*n* 36)

Positive impact on traditional/subsistence food access	Code definition	Discussed in # of interviews (*n* 36)	%
Sharing food	Sharing traditional/subsistence foods positively impacts access to them	10	28
Sharing food with Elders	Sharing traditional/subsistence foods with Elders increased access to them for that demographic	5	14
More traditional/subsistence foods	Respondent reported more access to traditional/subsistence foods during the pandemic	4	11
More use of traditional medicine	More use of traditional medicine reported due to the pandemic, including stinkweed and traditional foods as medicine	4	11
Negative impact on traditional/subsistence food access	Code definition	# (%) of interviews	
Restricted travel	Travel restrictions to the remote community from urban and other remote communities reduced capacity to obtain subsistence/traditional foods	10	27
Less traditional/subsistence foods	Respondent reported limited access to traditional/subsistence foods due to the pandemic in general (*n* 7) and less for Elders specifically (*n* 2)	9	25
Limited processing/storing foods	Fewer individuals and/or supplies available to process (*n* 4)/store (e.g. jar) (*n* 4) foods	8	22
Fewer people to hunt/fish/pick	Fewer hunters, fishers and other harvesters present in the community to obtain foods	7	19
Fewer economic resources	Fewer economic resources were available for gas and other supplies needed to obtain foods	2	6
Decrease in available knowledge	Due to the inability to travel or gather in large groups, knowledge related to food procurement, processing and storage was less available	1	3

Subsistence/traditional food access was impacted by a number of factors, most notably by restricted travel that prevented individuals from travelling to remote communities from other remote or urban communities to help with hunting, fishing and/or gathering activities. A Tribal leader and Elder described the intricacies of these changes in travel and subsistence in the following quote:

*We’re used to going out as family groups prior to COVID to either hunt or gather. Now that we have COVID with us, it is limiting the # number of hunters that are within your immediate family, or if you’re used to going on a boat upriver to pick greens/berries… [It has] limited opportunities for younger folks who don’t live in the community, but who want to help.*

*A lot of family in [remote Alaska] was hesitant to come to Anchorage… We as a family worked together to pay for the cooler of Native foods to come to Anchorage for those of us who didn’t want to risk transmission to the community. Awkward to not be able to go home to help with cutting fish, pick berries myself, help them fill up their freezers, participate in the barter system.*

However, sharing subsistence/traditional foods was cited equally as frequently, with interviewees citing that this positively contributed to subsistence/traditional food access despite the challenges brought by the COVID-19 pandemic. This theme, as well as sharing with Elders, is demonstrated by the following quote from an Alaska Native Elder living in a rural hub community:

*There’s still that tradition where people are still helping each other out in the village. My friend will ask me if I want any kind of Native food. We still give to our Elders. We leave it outside our doors. There are young people that go out hunting and bring food to the Elders first, and then they give out food to everyone else. I don’t feel like people had a harder time getting Native food this year.*

### State-wide online surveys of remote Alaska community members

The first round of online surveys (*n* 107) represents respondents from thirty-nine remote Alaskan communities (*n* 107), and the second round of surveys (*n* 508) represents respondents from 106 remote Alaskan communities (Table 4). The greater number of respondents for survey 2 was due to more prolonged advertisement and additional outreach to community organisations who shared the survey link on their online platforms as well. Respondents in both surveys were majority female, 25–54 years old and most had had some college, Associate’s or vocational programme training or higher education and had an annual household income of between $10 000–69 999. There was also a slight majority in Alaska Native participation, followed by white individuals, but also included respondents who identified as African American, Asian, Latino or more than one race/ethnicity. There were no statistically significant differences between the survey 1 and survey 2 samples for age, sex, annual household income, educational attainment or race/ethnicity.

**Table 4:** Summary of survey respondent demographic characteristics

	Weighting variables	Survey 1 (*n* 107)			Survey 2 (*n* 508)		
	Prop. in remote AK	No. of subjects in sample	Unweighted prop.	Weighted prop.	No. of subjects in sample	Unweighted prop.	Weighted prop.
Sex							
Male	54·3	20·0	18·7	48·0	121·0	23·8	53·5
Female	45·7	87·0	81·3	52·0	387·0	76·2	46·5
Age (years)	–	–	–	–	–	–	–
18–24	11·5	8·0	7·5	5·4	41·0	8·1	10·7
25–54	53·8	75·0	70·1	60·8	324·0	63·8	56·5
55–64	19·0	14·0	13·1	16·9	89·0	17·5	18·2
65+	15·7	10·0	9·3	16·9	54·0	10·6	14·6
Race/ethnicity	–	–	–	–	–	–	–
African American	1·7	1·0	0·9	0·1	6·0	1·2	1·4
Alaska Native	41·8	52·0	48·6	42·6	314·0	61·8	43·4
Asian	8·6	2·0	1·9	4·1	10·0	2·0	6·4
White	42·0	43·0	40·2	49·3	128·0	25·2	42·5
Latino	4·5	0·0	0·0	0·0	1·0	0·2	1·2
More than one	5·3	9·0	8·4	3·9	49·0	9·6	4·1
Education	–	–	–	–	–	–	–
8th grade or less	–	0·0	0·0	0·0	1·0	0·2	0·3
Did not fish HS	–	2·0	1·9	1·0	17·0	3·3	2·9
HS or GED	–	10·0	9·3	16·3	143·0	28·1	23·0
Some college, Associate’s, or vocational programme	–	55·0	51·4	45·5	216·0	42·5	40·9
College degree, post-graduate or professional school	–	40·0	37·4	37·3	131·0	25·8	32·8
Annual income	–	–	–	–	–	–	–
< $10 000	–	6·0	5·6	2·6	76·0	15·0	11·7
$10 000–29 999	–	15·0	14·0	14·6	122·0	24·0	27·4
$30 000–49 999	–	21·0	19·6	16·3	76·0	15·0	10·8
$50 000–69 999	–	18·0	16·8	17·3	82·0	16·1	14·1
$70 000–89 999	–	14·0	13·1	13·0	55·0	10·8	11·9
$90 000 and over	–	31·0	29·0	33·7	90·0	17·7	23·0
Missing	*–*	2·0	1·9	2·4	7·0	1·4	1·0

**Fig. 1:**
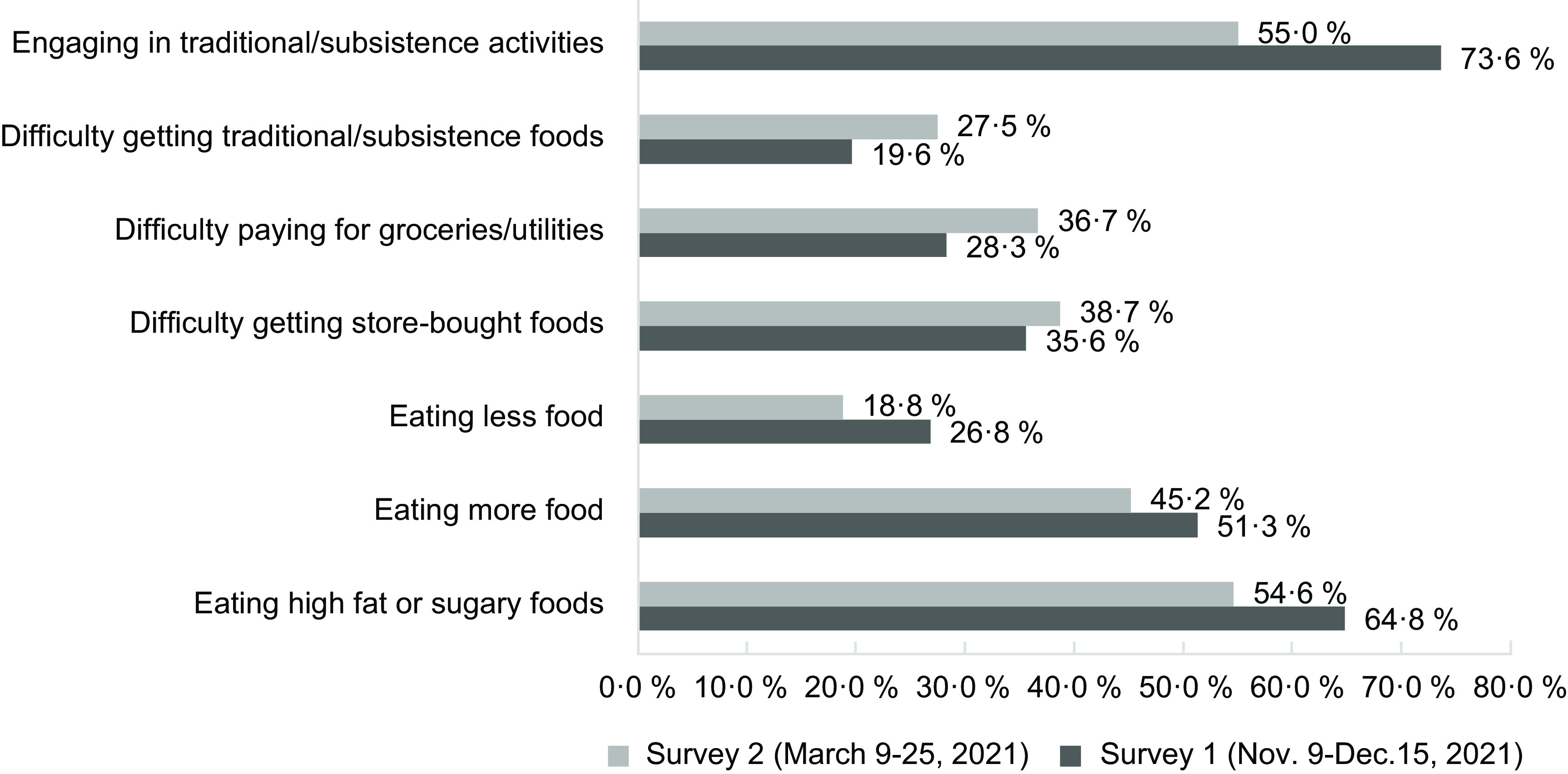
Impacts on food access due to the COVID-19 pandemic in remote Alaskan communities

Overall, there was a higher percentage of participants who reported food-related impacts in survey 1 compared with survey 2 regarding difficulty paying for groceries/utilities, difficulty getting store-bought foods and difficulty getting traditional/subsistence foods (Fig. 1). Notably, 27·5 % respondents in survey 1 reported having difficulty getting traditional/subsistence foods, which is 11·2 % less compared with the percentage of participants who reported having difficulty getting store-bought foods (38·7 %). A similar pattern is evident among survey 2 participants, with 19·6 % reporting difficulty getting traditional/subsistence foods compared with store-bought foods (28·3 %), an 8·7 % difference.

In contrast, the coping strategies to deal with the impacts of the pandemic were reported more frequently during survey 1 compared with survey 2 (Fig. 1). Almost 75 % respondents reported engaging in subsistence activities during survey 1, compared with slightly over half of respondents in survey 2. Eating patterns were also impacted, with the most common coping strategy in this category being eating high fat or sugary foods to cope with the pandemic, followed by eating more food in general, and finally eating less few in general.

## Discussion

The COVID-19 pandemic altered local and regional food environments and food-related behaviours in multifactorial ways^(1,2)^. The vast majority of studies on such impacts have focused on store-bought foods, making the present study distinct in that it also identifies several impacts of the pandemic on access to both traditional/subsistence food and store-bought foods in remote Alaskan communities. These changes have occurred alongside disruptions in other aspects of daily life, including restricted travel, altered behavioural outcomes and limited gatherings associated with the COVID-19 pandemic (LP Eichelberger, RL Fried, P Cochran and MB Hahn, unpublished results)^(25)^. Overall, this study demonstrates that store-bought food access was negatively impacted in multiple ways by the COVID-19 pandemic, which is in line with studies that identified and described pandemic-related supply chain disruptions^(1,26)^. In contrast, access to traditional/subsistence foods was shown to be less impacted in remote Alaskan communities.

The survey and KII findings of this study both support Remote Alaskan communities remained vulnerable to reduced food access during the pandemic, with 28–39 % of survey participants reporting difficulty paying for groceries or simply obtaining store-bought foods. Reasons for such difficulties were revealed in the KII analyses, which included travel restrictions, transportation/postal service disruptions and increased food prices. These documented experiences are similar to those reported in studies conducted in other rural and remote USA and international locations as well^(2,27–29)^. Prior studies have connected these food access barriers to decreased consumption of nutritious foods such as fruits and vegetables, indicating potential short- and long-term health impacts^(2)^.

Studies have found that the pandemic and related social isolation resulted in higher food consumption overall and an increase in high fat and sugary foods^(30,31)^. Similarly, survey results from this study showed that many more people ate more food (45–51 %) rather than less (19–27 %) after the beginning of the pandemic. Furthermore, more than half of individuals reported eating high fat and sugary foods, which is a higher percentage compared with those reported in other related studies conducted during the pandemic^(31–33)^. These changes in diet have been associated with subsequent weight gain, which points to the potentially increased need for public health intervention with regard to healthy eating and body weight in remote Alaska and beyond^(30)^.

Reported dietary changes and food access difficulties occurred alongside positive impacts to store-bought food access in communities. Key informants noted greater food assistance through churches, schools and food banks, as well as greater ease of grocery shopping through new delivery/phone-orders made available during the pandemic. Other studies also demonstrated greater access to food assistance programmes^(34)^, as well as a shift to less risky food purchasing behaviours such as shopping online^(35)^. Combined, these data indicate additional sources of resilience in remote Alaskan communities, some of which were supported through additional Federal, State and local funding, as well as adaptation of local grocery stores to meet the food needs of residents^(35)^.

In contrast to store-bought foods, our findings indicate that access to traditional/subsistence foods was less, and in some cases positively, impacted. Survey data indicated that 55–74 % of participants engaged in traditional/subsistence activities during the study period (surveys 1 and 2, respectively), and interview data indicate that sharing wild foods with Elders and others was common, and that there was more use of traditional medicines. These may be some of the reasons why fewer survey respondents reported difficulties getting traditional/subsistence foods compared with store-bought foods (9·2 % fewer in survey 1 and 8·7 % fewer in survey 2). A number of other additional factors could have contributed to this discrepancy as well, including comparatively less reliance on transportation and shipping from outside the community, more time available for subsistence activities because of travel restrictions, loss of job or reduced hours, working from home and participating in traditional/subsistence activities as a coping strategy.

Research has shown that diets high in wild foods help meet national dietary recommendations in terms of protein, fat and micronutrient intake and are associated with improved metabolic health outcomes^(36,37)^. This study shows that this unique aspect of rural Alaska food environments also likely contributed to more consistent access to foods despite supply chain disruptions, a finding demonstrated by this study in which participants cited fewer disruptions to traditional/subsistence food access in comparison to store-bought foods. Studies prior to the pandemic highlighted the importance of local wild foods for mitigating food security in rural Arctic communities, and this study indicates those protections persisted through the pandemic as well^(18,38)^. Although some barriers to accessing wild foods were cited, such as travel restrictions limiting the number of people who could contribute to such efforts, as well as fewer economic resources to support these activities, continued access to wild foods may have protected rural residents from more severe food insecurity and poor health outcomes during the COVID-19 pandemic^(39,40)^.

While this study is the only state-wide study assessing the impact of the COVID-19 pandemic on both traditional/subsistence and store-bought food access, it does have limitations. First, we are reporting on a state-wide scale in only remote communities, so we are not detailing impacts on urban food environments or differences in trends or patterns across different remote communities, regions or groups thereof. Second, we would like to note that the thirty-six KII of residents of remote Alaskan communities, while few in number, provide a greater depth of perspective than the survey data. As has been noted previously, individuals with the life experiences captured here can advance our understandings of phenomena, while not seeking to generalise such perspectives to the greater population^(41)^. Therefore, we strategically included these in-depth interviews because they provide critical insight into how the COVID-19 pandemic impacted food access beyond what was possible to capture in the online surveys.

In addition, the 615 online survey participants were a convenient, non-representative sample, and although we corrected analyses somewhat with statistical weighting methods, the sample was still limited to individuals who had access to Facebook and/or email. Finally, some of the reported differences in food access and food-related coping activities between the first and second surveys were within the margin of error for the sample size. However, the consistency in responses across survey questions and further triangulation with the qualitative interview data provide additional confidence in our observations regarding the impact of the pandemic on food access in remote Alaskan communities.

The results of this study illustrate that the specific geographic, socio-political and cultural contexts of remote Alaskan communities provide sources of both resilience and risk within the broader context of the COVID-19 pandemic. As one interviewee stated, ‘Remoteness was a blessing, but also a potential downfall’. These data demonstrate that the pandemic had significant impacts on both store-bought and traditional/subsistence food access in remote Alaskan communities, but also that locally available wild foods provided a potential buffer against external disruptions in the availability of store-bought foods and served as a broader coping mechanism during times of stress. These food access dynamics may have important implications for long-term health outcomes as well, including cardiometabolic health^(42)^. Furthermore, these data provide additional support to prior studies showing how globalised food distribution chains are more likely to be interrupted and become disordered in comparison to local foods, and that the need for local food alternatives is becoming increasingly evident in light of this pandemic and as we look for potential alternatives to food access in future national and global health and environmental-related crises^(43,44)^.
